# Role of platelet-rich fibrin in soft and hard tissue healing after impacted third molar surgery: A triple-blind split-mouth randomized controlled clinical trial

**DOI:** 10.34172/joddd.41122

**Published:** 2024-12-14

**Authors:** Ifra Iftikhar, Sanjay Singh, Ashu Bhardwaj, Mandeep Kaur, Priyanshu Kumar Shrivastava, Nitika Monga, Deborah Sybil

**Affiliations:** ^1^Department of Oral and Maxillofacial Surgery, Faculty of Dentistry, Jamia Millia Islamia, New Delhi, India; ^2^Department of Periodontics, Faculty of Dentistry, Jamia Millia Islamia, New Delhi, India; ^3^Department of Oral Medicine and Radiology, Faculty of Dentistry, Jamia Millia Islamia, New Delhi, India; ^4^Faculty of Dentistry, Jamia Millia Islamia, New Delhi, India; ^5^Division of Non-Communicable Diseases, Indian Council of Medical Research Headquarters, New Delhi, India; ^6^Department of Oral and Maxillofacial Surgery, Faculty of Dentistry, Jamia Millia Islamia, Maulana Mohammad Ali Jauhar Marg, Jamia Nagar, New Delhi-110025, India

**Keywords:** Healing, Pain, Platelet-rich fibrin, Surgery, Third molar

## Abstract

**Background.:**

Platelet-rich fibrin (PRF) enhances tissue healing by releasing essential growth factors. Surgical extraction of deeply impacted mandibular third molars poses a common challenge, often leading to significant defects at the distal root of the second molar. This study explored the role of PRF in soft and hard tissue healing after surgical extraction.

**Methods.:**

This triple-blind, split-mouth, randomized controlled trial involved patients with bilateral impacted mandibular third molars. Single-stage surgical extraction was performed, and PRF was applied at one site while the other served as the control. Plaque index (PI), sulcus bleeding index (SBI), clinical attachment levels (CALs), postoperative pain, edema, tenderness, sensitivity, and bone level were assessed on day 1, day 3, first week, and first, third, and sixth months.

**Results.:**

Sixty-four (34 males and 30 females) patients were found eligible for assessment. The test group exhibited a significant decrease in mean pain scores compared to controls (*P*<0.001), notably resolving by one month. Edema scores were significantly lower in the test group at all intervals up to one month (*P*=0.045). Tenderness showed a significant difference at one week (*P*=0.001), resolving by three months. No significant hard tissue changes were noted (*P*=0.825).

**Conclusion.:**

Significant benefits over postoperative pain, bleeding, tenderness, and initial sensitivity underscored the importance of PRF in soft tissue healing following impacted mandibular third molar extraction. However, no improvement in bone height outlined its limited potential in hard tissue regeneration over exposed root surfaces of the mandibular second molar.

## Introduction

 Extraction of deeply impacted mandibular third molars may cause significant defects at the distal root of the second molar. Many patients report extreme sensitivity in the post-extraction area, which is attributed to the cemental exposure of the distal root of the second molar. This can disturb the patient, reducing the quality of life as it hampers normal eating and drinking. One of the latest achievements in dentistry is the use of platelet-rich fibrin (PRF) to improve the repair and regeneration of soft and hard tissues after surgical procedures. PRF represents a new step in the platelet gel therapeutic concept with simplified processing minus artificial biochemical modification.^1^

 Studies have demonstrated that viable platelets in PRF release six growth factors, namely, platelet-derived growth factor (PDGF), vascular endothelial growth factor (VEGF), transforming growth factor (TGF), insulin-like growth factor (IGF), epithelial growth factor (EGF), and recombinant human basic fibroblast growth factor (bFGF).^2^ These growth factors stimulate cell proliferation, angiogenesis, and the formation of new extracellular matrix, promoting tissue regeneration and repair. Additionally, PRF possesses antimicrobial properties and a three-dimensional fibrin architecture, further contributing to its favorable impact on wound healing.^3^ Li et al^4^ reported the effectiveness of PRF in enhancing alveolar bone augmentation. In contrast, several clinical investigations failed to demonstrate statistically significant advancements in alveolar bone regeneration with PRF.^5-8^ This conflicting evidence underscores the importance of our study, contributing to the ongoing debate regarding the impact of PRF on hard tissue regeneration.

 A recent systematic review by Millard et al^9^ emphasized the need for additional randomized controlled trials and standardization of protocols to draw definitive conclusions regarding the efficacy of PRF in alveolar bone regeneration. A meta-analysis focusing on PRF as an adjunct to bone grafts in alveolar bone augmentation concluded that the current evidence was limited to sufficiently support the use of PRF.^4^ The necessity for well-designed RCTs employing consistent PRF formulations to validate their findings was further recommended.

 Although PRF has been investigated for its potential in assessing osseous regeneration and soft tissue healing in human mandibular third molar extraction sites, its efficacy over exposed root surfaces of the second molar and its influence on cortical bone formation has not been thoroughly studied. Therefore, this randomized controlled trial systematically evaluated the osseous regeneration capacity of PRF, specifically in the formation of cortical bone over exposed root surfaces of mandibular second molars following the surgical extraction of impacted third molars. Furthermore, this study was prompted by the need to broaden our understanding of PRF’s application and its potential impact on periodontal soft tissue healing in this unique clinical context.

## Methods

###  Study design and ethics

 The study was conducted in the Department of Oral and Maxillofacial Surgery at a tertiary dental care center. The null hypothesis for the present study was the addition of PRF to the mandibular third molar extraction site does not augment soft and hard tissue healing. Ethical clearance was obtained from the Institutional Ethical Committee (1/10/300/JMI/IEC/2020), and a pilot study comprising 25 patients was performed before this study.^10^ The proposed study was registered as a clinical trial with CTRI (Clinical Trial Registry of India) under reference number CTRI/2020/11/028865. This split-mouth randomized controlled trial was performed in accordance with the CONSORT guidelines.^11^ All the patients fulfilling the inclusion criteria were explained about the study, and consent was obtained. All the study participants were given a unique ID number for identification.

###  Sample size calculation

 Sample size calculation was done with an 0.01 level of significance and 95% power. A standard deviation of 0.5 mm was taken from our previously published pilot study.^10^ A sample size of n = 64 was calculated to achieve the desired comparison.

###  Participants

 Each patient was treated using two therapeutic approaches, yielding two different groups. One site acted as control, and the other, where fresh autologous PRF was placed in the socket, was the test site. Eligibility criteria were defined as follows:

###  Inclusion criteria

1. Patients 18‒50 years of age
2. Patients with complete bilateral impacted mandibular third molars
3. Radiographic bone loss of > 3 mm distal to the second molar

###  Exclusion criteria

1. Medically compromised and pregnant patients
2. Patients with abnormal platelet counts ( < 200 000/mm^3^)
3. Patients with hopeless or missing second molars
4. Patients with smoking and tobacco habit(s)
5. Patients with a history of medications three months before the intervention, which might interfere with wound healing

###  Outcome measures

 The primary outcome was to assess bone regeneration over exposed root surfaces of second mandibular molar teeth. Secondary outcome measures included postoperative pain, tenderness, edema, sensitivity at the operated site, plaque index (PI), sulcular bleeding index, and clinical attachment levels (CALs). Patients underwent oral prophylaxis one week before the surgery. Baseline characteristics, including PI, sulcus bleeding index (SBI), and CALs, were recorded just before the surgery, and immediate postoperative CBCT radiographs were taken to assess the bone loss distal to the second molar tooth. Pain and tenderness, sensitivity index, and edema scales were recorded postoperatively on the first and third day, first week, and first, third, and sixth month to evaluate soft tissue healing. A visual analog scale (VAS) was used to assess pain and tenderness postoperatively. The sensitivity index and edema scale are described in Table 1.^10^ Area-specific PI and SBI were recorded after one week and at the end of the first, third, and sixth months postoperatively. CAL was measured at three and six months postoperatively. A second CBCT was recorded at the six-month follow-up to check for bone levels distal to the second molar and compare it with the first recorded CBCT. Additional outcomes were to observe any complications or adverse events following intervention.

**Table 1:** Scoring criteria for edema and sensitivity

**Score**	**Parameter**	
	**Edema **	**Sensitivity **
0	No edema	Negative response to all the three stimuli
1	Either intraoral or extraoral edema	Positive response to any one of the three stimuli
2	Both intraoral and extraoral edema	Positive response to any two of the three stimuli
3	Not applicable	Positive response to all the three stimuli

###  Intervention

 Blood samples were obtained from the same patient before the surgery for PRF preparation. PRF fabrication was done for each sample using the previously described Choukroun Technique.^2^ A single-stage surgical extraction of bilateral mandibular third molars was performed under classical inferior alveolar nerve block for all the patients by the same surgeon. Ward’s incision was given distal to the second molar, and a full-thickness flap was raised to expose the alveolar bone. The decision on bone removal was taken by the surgeon on a need-basis. After delivery of the tooth from its socket, an immediate CBCT assessment was performed to determine eligibility concerning more than 3 mm of radiographic bone loss. The test site was packed with freshly prepared PRF, whereas the control site received no such preparation. Primary wound closure was achieved using an interrupted suturing technique. Standard postoperative instructions were given.

###  Randomization and blinding

 Randomization was done by the minimization technique. A computer-generated binary code system randomly determined the test and control sites. Surgical extraction was performed by another investigator, while the remaining investigators conducting clinical and radiographic assessments were blinded to the test and control sides to avoid bias.

###  Statistical analysis

 The data were organized and submitted for statistical analysis using SPSS 10.0 (SPSS Inc., Chicago, IL, USA). Repeated-measures ANOVA and post hoc Bonferroni tests were applied for intragroup comparison at different time frames. For intergroup comparison between test and control sites, a paired t-test was applied. *P* < 0.05 was considered significant.

## Results

**Figure 1:**
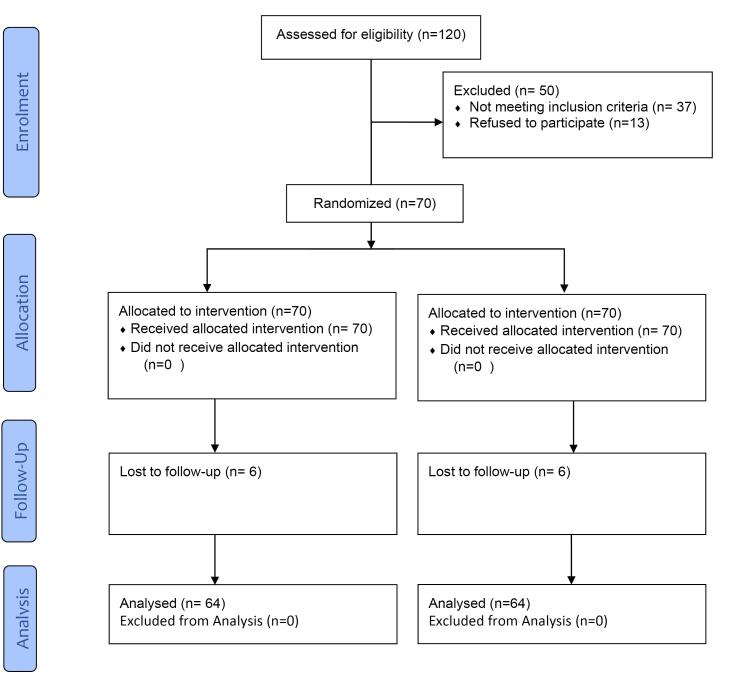
CONSORT flow diagram according to the criteria adopted in this clinical trial

 A total of 120 patients were assessed for eligibility to account for attrition bias, 70 of which were selected. Six patients were lost to follow-up, and 64 were evaluated (Figure 1). The male-to-female ratio was 34:30. The mean age of the participants was 35.82 (SD 5.77), ranging from 24 to 47 years.

 The mean PI exhibited a significant increase one week after surgery at both control and test sites, followed by a marginal decrease at one month and a subsequent rise in the test group at three months. Notably, a statistically significant difference in the mean PI between the test and control groups was observed at six months. The mean SBI demonstrated a statistically significant difference at one week (*P* < 0.001) between the test and control groups, with subsequent time intervals showing non-significant differences. CAL exhibited no statistically significant differences at all the observation time intervals (3 months; *P* = 0.261). No significant improvement in CAL was noted from baseline until six months in both the test and control groups (Table 2).

**Table 2:** Intergroup comparison of plaque index, sulcus bleeding index, and clinical attachment levels

		**Plaque index**			**Sulcus bleeding index**			**Clinical attachment levels **		
		**Mean**	**Standard deviation**	***P*** ** value**	**Mean**	**Standard deviation**	***P*** ** value**	**Mean**	**Standard deviation**	***P*** ** value**
Baseline	Test	0.5625	0.68718	0.131, NS	0.2031	0.62182	0.251, NS	1.6781	0.47525	0.200, NS
	Control	0.4675	0.61399		0.2656	0.57022		1.6313	0.53833	
1 week	Test	1.1250	0.54917	0.196, NS	0.2344	0.42696	< 0.001, S	-	-	-
	Control	1.0156	0.41756		0.7656	0.52681		-	-	
1 month	Test	0.6250	0.60422	< 0.001, S	0.2098	0.42696	0.180, NS	-	-	-
	Control	1.0156	0.41756		0.3438	0.47871		-	-	
3 months	Test	0.9375	0.63932	0.999, NS	0.0000	0.0000	-	1.3938	0.85484	0.261, NS
	Control	0.9375	0.50000		0.0000	0.0000		1.4375	0.83713	
6 months	Test	0.6250	0.60422	< 0.001, S	0.0000	0.0000	-	1.4597	0.63730	0.161, NS
	Control	1.0156	0.41756		0.0000	0.0000		1.703	0.81234	

S: Significant; NS: Not significant

 A statistically significant difference was noted in the mean pain scores between the test and the control groups throughout subsequent follow-ups (*P* < 0.001). Within the test group, mean pain scores exhibited no significant alteration until day 3, followed by a noteworthy reduction at one week and complete resolution by one month (post hoc pairwise comparison: day 1, day 3 > week 1 > months 1, 3, and 6). Mean edema scores between the test and control sites were also significantly different at all follow-up intervals up to one month (*P* = 0.045). However, no edema was discernible in either group during later follow-up periods (mean = 0.000, SD = 0.0000). Progression of edema as per post hoc pairwise comparison was days 1 and 3 > week 1 > months 1, 3, and 6. Intergroup comparison of mean tenderness scores between test and control sites revealed a statistically significant difference at the 1-week follow-up (*P* = 0.001), while no significant distinctions were noted at day 1 (*P* = 0.641), day 3 (*P* = 0.829), and subsequent follow-ups (1 month; *P* = 0.321). From 3 months onwards, tenderness was absent in both groups. Initially, sensitivity was found to be significantly less at the test sites (*P* < 0.001), followed by no significant difference during later follow-up intervals (*P* = 0.159). Table 3 illustrates comprehensive data on intergroup comparison of postoperative pain, edema, tenderness, and sensitivity. Concerning the hard tissue changes, there was no significant improvement in bone height from baseline to 6 months in both groups, and no significant difference was noted between the test and control groups (*P* = 0.825) (Table 4). A few minor complications were noted in the control group, including dry socket (n = 4; 6.25%) and postoperative bleeding (n = 2; 3.125%). No adverse events or complications were noted at the test site.

**Table 3:** Intergroup comparison of postoperative pain, edema, tenderness, and sensitivity

		**Pain**			**Edema**			**Tenderness**			**Sensitivity**		
		**Mean**	**Standard deviation**	***P*** ** value**	**Mean**	**Standard deviation**	***P*** ** value**	**Mean**	**Standard deviation**	***P*** ** value**	**Mean**	**Standard deviation**	***P*** ** value**
Day 1	Test	3.1875	1.13913	< 0.001, S	1.3750	0.62994	< 0.001, S	1.1094	0.44068	0.641, NS	0.1875	0.39340	< 0.001, S
	Control	3.7344	1.28936		1.9688	0.89031		1.2031	0.39308		0.5156	0.53429	
Day 3	Test	3.1875	1.24563	0.001, S	1.3500	0.87287	< 0.001, S	1.0875	0.39340	0.829, NS	0.0781	0.27049	< 0.001, S
	Control	3.6406	1.23754		2.3906	0.74785		1.1406	0.44292		0.4063	0.49501	
1 week	Test	0.2031	0.89073	< 0.001, S	0.0313	0.17537	< 0.001, S	0.3438	0.54098	0.001, S	0.1094	0.31458	0.096, NS
	Control	1.5156	1.26214		1.5625	0.70991		0.7063	0.60994		0.1875	0.39340	
1 month	Test	0.0000	0.00000	0.001, S	0.0000	0.00000	0.045, S	0.0000	0.0000	0.321, NS	0.1719	0.38025	0.531, NS
	Control	0.1563	0.36596		0.06250	0.24398		0.0156	0.12500		0.2031	0.40551	
3 months	Test	0.0000	0.00000	-	0.0000	0.00000	-	0.0000	0.0000	-	0.2500	0.43644	0.484, NS
	Control	0.0000	0.00000		0.0000	0.00000		0.0000	0.0000		0.2813	0.45316	
6 months	Test	0.0000	0.00000		0.0000	0.00000		0.0000	0.0000		0.2031	0.40551	0.159, NS
	Control	0.0000	0.00000		0.0000	0.00000		0.0000	0.0000		0.2656	0.44516	

S: Significant; NS: Not significant.

**Table 4:** Intergroup comparison of mean gain in Bone height

**Mean bone level**		**Mean**	**N**	**Standard deviation**	***P*** ** value**
At baseline	Test	3.7031	64	0.42350	0.39, NS
	Control	3.6609	64	0.50603	
6 months	Test	3.7594	64	0.43121	0.825, NS
	Control	3.7469	64	0.37627	

S: Significant; NS: Not significant.

## Discussion

**Figure 2:**
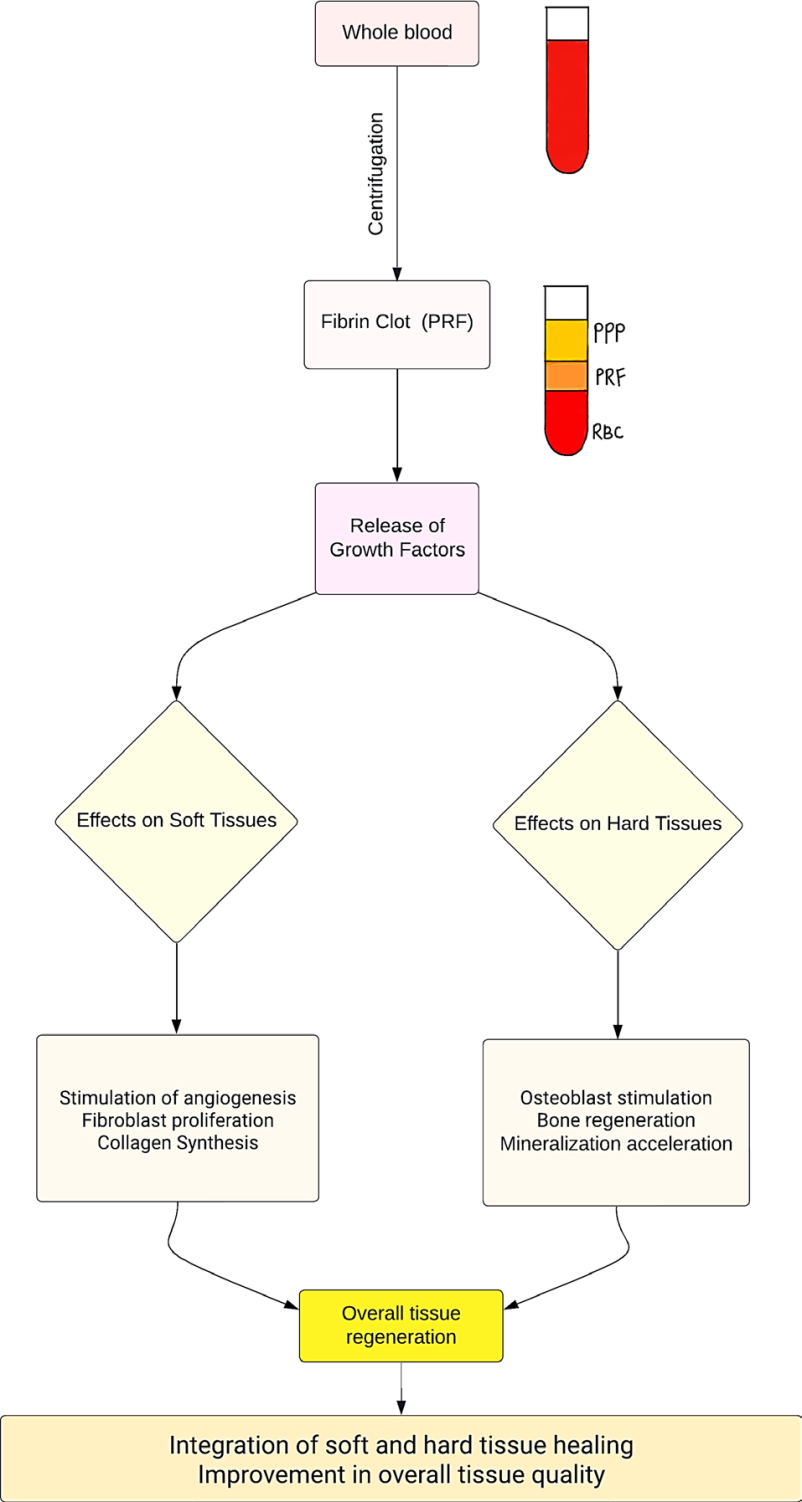
Flowchart illustrating the role of platelet-rich fibrin in soft and hard tissue healing

 Exploring platelets’ regenerative potential dates back to 1974 when Ross et al^12^ conducted seminal research on a platelet-dependent serum factor that stimulated the proliferation of arterial smooth muscle cells in vitro. This investigation elucidated the release of growth factors from platelets, marking a pivotal moment in understanding their therapeutic capabilities. PRF, identified as a platelet derivative, serves as a matrix where platelets, cytokines, growth factors, and cells are entrapped and gradually released over time. Upon activation, platelets confined within the fibrin matrix stimulate a mitogenic response by releasing growth factors that complement wound healing.^13^ Figure 2 illustrates the role of PRF in facilitating both soft and hard tissue healing. Consequently, the observed soft and hard tissue changes attributed to PRF can be extrapolated to the periodontium following surgical procedures, emphasizing the need to compare and assess the healing status of the periodontium with and without the assistance of autologous platelet concentrates.

 Notably, in the current trial, PRF was found efficient in soft tissue healing with statistically significant differences across several parameters, including postoperative pain, edema, tenderness, and initial sensitivity. No incidence of dry sockets in the test group also supports the healing capabilities of PRF. Another meta-analysis supported these findings wherein local application of PRF during lower third molar extraction prevented postoperative complications and significantly relieved the pain and swelling. However, significant differences were not found in soft tissue healing between the PRF and non-PRF groups.^14^ Another meta-analysis, including nineteen randomized controlled trials, confirmed decreased incidence of dry sockets and postoperative pain following PRF application and better soft tissue healing.^15^ Nonetheless, hard tissue changes were not found significant in the current study, with non-significant differences in bone height over the exposed root surfaces of second molar teeth within six months, both within the test group and in comparison between the test and control groups. This outcome was consistent with the findings from a systematic review where PRF failed to demonstrate any beneficial role in bone healing following mandibular third molar extraction.^16^

 Li et al^3^ showed that PRF enhances osteogenic lineage differentiation of alveolar bone progenitors more than of periodontal progenitors by augmenting osteoblast differentiation, RUNX2 expression, and mineralized nodule formation via its principal component, fibrin. They also documented that PRF functions as a complex regenerative scaffold, promoting tissue-specific alveolar bone augmentation and surrounding periodontal soft tissue regeneration via progenitor-specific mechanisms. However, the role of platelet concentrates in bone regeneration is weakly supported. A significant concern identified was the lack of nutrients and oxygen supplies to the cells located deep in the implanted scaffold.^17^ Another hypothesis suggested for the limited role of PRF in bone regeneration is the release time. The release of growth factors from PRF has been reported to be up to 7 days for most of them,^18^ whereas bone formation takes weeks to months and requires controlled release of growth factors for osteogenic differentiation. This explains the inefficiency of PRF in bone regeneration. However, this claim is unsupported and requires additional investigations.

 The increase in PI one week after surgery in both groups is a common finding following oral surgery due to inefficient hygiene around the postoperative site. However, the subsequent differences in PI between the test and control groups at six months highlight the potential long-term benefits of PRF in maintaining oral hygiene, which could be attributed to either patients’ oral hygiene or other contributing factors such as increased clinical attachment loss and pocket depth at control sites. No significant differences in the CAL were seen between the control and test sites and without any significant improvement at subsequent follow-ups, suggesting the minimal role of PRF in re-gaining gingival attachment following extraction of third molars. A meta-analysis evaluating the effectiveness of PRF membranes on root coverage, CAL, or keratinized mucosa width in treating gingival recessions found no significant changes.^19^ Conversely, a clinical trial noted a greater reduction in probing depth, gain in CAL, and greater intra-bony defect fill with autologous PRF treatment compared to open flap debridement alone.^20^ Bleeding index showed a significant difference between the test and control sites one week postoperatively, which signifies the beneficial role of PRF in early resolution of gingival inflammation.

 PRF has diverse applications in dentistry beyond the scope of this study. It can serve as a scaffold or gel for tissue regeneration, wound healing, pulp revascularization procedures, and bone augmentation. Its potential applications extend to various dental procedures, including implantology, periodontal surgeries, and oral and maxillofacial surgeries.^21^ While this study was meticulously designed and controlled for various factors, confounding variables such as patient compliance with postoperative instructions, minor variations in surgical technique, and individual healing capacities could still influence the outcomes. Moreover, the external validity of the trial findings may be limited by factors such as patient demographics, variations in surgical protocols, and specific conditions present in the study population. Replicating the study in diverse settings and populations would enhance the generalizability of the findings. Carry-over-effects commonly observed in split-mouth trials could further affect the study’s outcomes. This study highlighted the benefits of PRF in terms of reduced postoperative pain and bleeding with PRF and improved soft tissue healing. Additionally, complications noted in the control group, including dry socket and postoperative bleeding, emphasize the importance of weighing the benefits of PRF against potential demerits.

## Conclusion

 This split-mouth randomized trial noted valuable insights into the positive effects of fresh autologous PRF as evidenced by the reduction in postoperative pain, bleeding, tenderness, and initial sensitivity following impacted mandibular third molar extraction. These results and reduced postoperative complications with the use of PRF support its effectiveness in soft tissue healing over exposed root surfaces of the mandibular second molar. However, PRF exhibited a restricted role in enhancing radiographic bone height and CAL. Despite its limited role in hard tissue healing, the ease in preparation, high safety rate, and negligible reported adverse events promote PRF as an attractive option for application over exposed root surfaces of the mandibular second molar following surgical disimpaction of the mandibular third molar. However, carefully considering patients’ medical history and economic factors is necessary when applying these findings to clinical practice. Further parallel-arm trials, including larger sample sizes, are warranted to establish the applicability and long-term efficacy of PRF in soft and hard tissue healing over exposed root surfaces of the mandibular second molar.

## Acknowledgments

 This project was carried out in accordance with the Indian Council of Medical Research and Good Clinical Practice guidelines.

## Competing Interests

 None.

## Data Availability Statement

 The data supporting the findings of this study are available from the corresponding author upon reasonable request.

## Ethical Approval

 Ethical clearance was obtained from the Institutional Ethical Committee (1/10/300/JMI/IEC/2020) dated 29/10/2020.
